# Bring the life stages into the domain of basic and clinical pharmacology

**DOI:** 10.3389/fphar.2022.923016

**Published:** 2022-12-13

**Authors:** Lan Yao, J. Carolyn Graff, Lotfi Aleya, Yan Jiao, Weikuan Gu, Geng Tian

**Affiliations:** ^1^ Department of Orthopedic Surgery and BME-Campbell Clinic, University of Tennessee Health Science Center, Memphis, TN, United States; ^2^ College of Nursing, University of Tennessee Health Science Center, Memphis, TN, United States; ^3^ Chrono-Environnement Laboratory, UMR CNRS 6249, Bourgogne Franche-Comté Université, Besançon Cedex, France; ^4^ Research Service, Memphis VA Medical Center, Memphis, TN, United States; ^5^ Department of Pharmaceutical Sciences, University of Tennessee Health Science Center, Memphis, TN, United States; ^6^ Shandong Technology Innovation Center of Molecular Targeting and Intelligent Diagnosis and Treatment, Binzhou Medical University, Yantai, Shandong, China

**Keywords:** clinical trial, drug, menopause, medicine, puberty

## Abstract

Completely distinct physiological conditions and immune responses exist among different human life stages. Age is not always consistent with the life stage. We proposed to incorporate the concept of the life stages into basic and clinical pharmacology, including clinical trials, drug labels, and drug usage in clinical practice. Life-stage-based medical treatment is the application of medicine according to life stages such as prepuberty, reproductive, and aging. A large number of diseases are life-stage-dependent. Many medications and therapy have shown various age effects but not been recognized as life-stage-dependent. The same dosage and drug applications used in different life stages lead to divergent outcomes. Incorporating life stages in medicine and drug usage will enhance the efficacy and precision of the medication in disease treatment.

## 1 Introduction-The unclarified issue in basic and clinical pharmacology

While gender and ethnic differences have been incorporated in basic and clinical pharmacology, the life stage has not been well defined as a category in personalized medicine. The reason is that an individual’s age is not an accurate measurement for a customized application of diagnosis and treatment. For example, for two 50-year-old females, one may be in the postmenopausal stage while the other may not reach menopause. Similarly, for two boys at the age of 13, one may have reached puberty while the other may not. In practice, many health professionals plan clinical trial protocols according to the age of the participants (Al-Sahab et al., 2012).

We proposed to use the life stage as one of the categories to improve therapeutic treatment and personalized medicine. We hypothesize that using the life stage as one of the parameters of a study enables a more complete understanding of the physiological and pathological characterization of a study subject in clinical studies. The life stage of humans can be divided into pre-birth, prepuberty, reproductive, and post-reproductive or aging stages (Gu, 2022a; Gu, 2022b). Accordingly, the transitions from one stage to the next would be studied and defined (Gu, 2022b). Instead of only by age, physiological changes such as body features, puberty, menopause, and andropause can be used to define the life stages. Measurement of internal physiological changes such as the hormone level and other related proteins or gene expression levels may be utilized for such a consideration (Gu, 2022a; Gu, 2022b). The physiological characteristics in each life stage need to be completely understood to apply the proper dose of medication and therapy. Gender in the life stage should be defined and integrated into clinical trials and studies. Until now, barriers in personalized medicine are partially caused by the limited understanding of the interaction between life stages and medication effectiveness. Therefore, we introduced the concept of life stages in the life cycle where the practice of medicine may be improved.

## 2 Policy options and implications-life stages and age differ significantly

### 2.1 The life stages in the life cycle in different organisms

From the simplest one-cell creatures to the complex life cycle of animals and humans, the features of different life stages are distinct (Gu, 2022a). Reviewing these differences enables us to understand the differences of mechanisms in the life stages and life cycles.

**FIGURE 1:**
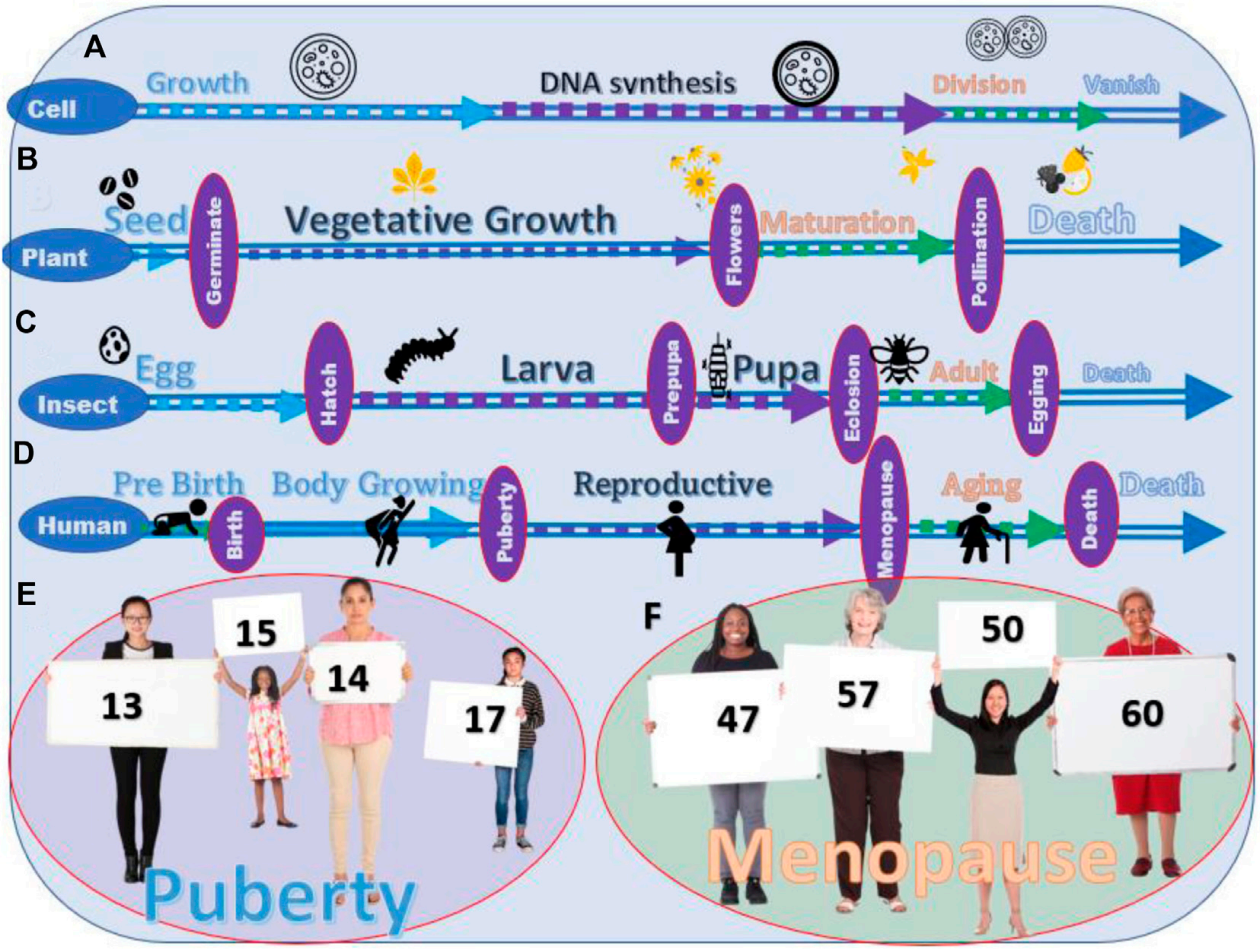
Life stages of different living creatures. **(A)** The stages of the life cycle of a cell. **(B)** Stages of the life cycle of crops and vegetables. **(C)** Four stages of the life cycle of most insects. **(D)** Stages of the life cycle of humans. **(E)** Variation in the age of puberty among young people. **(F)** Variation in the age of menopause among the human.

Although phenotypically invisible, a life cycle, the series of changes in the life of a simple organism, including reproduction, can be divided into three main stages, growth, DNA synthesis, and division (the death of the original cell) (Figure 1A). Similar patterns occur in plants after seeding, vegetative growth, flowering and polluting and seeding, and death of the plant (Figure 1B). The life of a butterfly or moth has very distinct phenotypic stages: fertilized egg→larva→pupa→adult→death (Figure 1C); however, they can also be divided into similar life stages, the growth (egg→larva→pupa), the reproductive (adult), the aging and death (death). Humans experience similar stages, body growth (prepuberty), reproductive (between puberty and menopause), and aging and death (postmenopause).

The complicated living creatures on earth arise from the simple lives. From the point of view of the life stage, humans are not unique. The life of humans is also divided into different life stages. The transition from one life stage to the other is visible in some organisms while not in others. Different life stages of humans are not shown in obvious phenotypic characteristics, such as between lava and pupa in insects, but the physiological changes can still be clearly defined (Figures 1D–F).

There is a large amount of morphological variation in these organisms while they have similar life stages in their life cycle (Thomas et al., 2001; Filatova et al., 2019). The variations of each life stage may be determined by environmental factors, such as living conditions, access to health services, and nutrition (supply and quality of food). Therefore, age may not accurately indicate therapeutic application of treatment in humans. However, life stages could better describe and measure the physiological and phenotypic changes.

### 2.2 Evidence of life-stage-dependent diseases

Diseases include childhood, adult, and aging diseases. These classifications are based on the age of disease development. A different classification of diseases may be prepuberty, reproductive, and aging (Gu, 2022a). Thus, the classification is based on the physiological changes of the life stages.

### 2.3 Childhood diseases should have been investigated considering prepuberty as one life stage

Many diseases that affect infants and children primarily are called childhood diseases, such as measles, mumps, acute lymphocytic leukemia (ALL), and childhood asthma. The definition of childhood is being a child, but the age interval of children and adolescents varies significantly, from 0 to 18. The timing of puberty as the indicator of physical maturity has been neglected.

Studies have shown the importance of menarche as an indicator of disease onset, including the causal inverse associations between puberty timing and risks for breast and endometrial cancers in women and prostate cancer in men (Mitra et al., 2012) and a strong association between age at menarche and the age at onset of bipolar and anxiety (Chakraborty et al., 2018). Unfortunately, the link between the life stages of growth and productivity is not established.

### 2.4 Period between puberty and menopause as the reproductive life stage and its impact on diseases

Although the reproductive life stage has not been marked as a particular time for diseases, evidence has shown its particular status on the diseases. A meta-analysis by the Collaborative Group on Hormonal Factors in Breast Cancer showed that during the period between puberty and menopause, women had a greater risk of breast cancer (Day et al., 2017). Similarly, in a study on the age of menarche and menopause with cardiovascular disease, diabetes, and osteoporosis in Chinese women, Qiu et al. reported that menarche and menopause history could be used as an indicator for women with an increased risk of developing cardiovascular disease and osteoporosis (Tondo et al., 2017). The incidence and pathological features of the diseases in the reproductive period differ from that of prepuberty and postmenopause (Collaborative Group on Hormonal Factors in Breast Cancer, 2012).

### 2.5 Menopause as the start of the aging life stage

Changes in physiological and immune systems are well known in postmenopausal women (Qiu et al., 2013; Ysrraelit and Correale, 2019). However, in the majority of clinical trials, menopause was not treated as an essential parameter. For example, searching the keyword “cancer” from the type of article as a clinical trial in the last 10 years from PubMed on 5 January 2022, we found 57,312 articles. By adding “menopause” as the keyword, only 897 articles were selected. These 897 articles are a small portion, even within the 7831 clinical trials of breast cancer. Instead, the majority of the clinical trials utilize age as one of the characterizations of the patients. When searching the article with the keywords “cancer” and “age,” 11,109 articles were found. The ages in different clinical trials are listed as investigation parameters varied in a wide range without clear rationale (Davis et al., 2015; Muka et al., 2016). Similar to the time of puberty, the age of menopause of a woman varies across different races, regions, times, and countries (Figure 1F). Ages, such as 55, 60, 65, and 70, do not correctly reflect the life stages of a patient (Davis et al., 2015; Muka et al., 2016).

### 2.6 The same treatment shows different effects at the different life stages

As different life stages have different physiological and pathological statuses, the same therapeutic treatment on different life stages has a different effect (Wang et al., 2017; Wang et al., 2021). A typical example is hormone replacement therapy (Lobo, 2017). Women benefit from treatment before puberty, including a reduction in the incidence and mortality of coronary heart disease. In contrast, treatment on postmenopausal women did not show such improvement but increased the risk of diseases (Lobo, 2017). However, the difference between different life stages is the hormones and the overall physiological status, including the immune systems (Hirokawa et al., 1992; Gameiro et al., 2010). More differences in the therapeutic applications should be identified when the life stage is used, like the characteristics of subjects in clinical trials.

For men, the research specifically focusing on drug dosages during different life stages has been largely neglected. On the other hand, the significant physiological changes during puberty and andropause have been clearly documented by a large number of publications. The age of puberty has been linked to disease and physiological abnormalities. For example, early puberty has been linked to men’s risk for type 2 diabetes (Ohlsson et al., 2020). Sex-related differences in adverse drug reactions have been well-documented (Watson et al., 2019). A significant change in puberty for men is masculinity which is associated with the changes in physiological features and level of androgen in men. Significant changes in health and response to the drug treatment are accompanied by masculinity at puberty (Liu et al., 2016; Lucas-Herald et al., 2016). Dosage of drugs based only on age does not fit well with the variation of ages of puberty and physiological and immune response of men.

### 2.7 Differences in age and trends of ages of life stages among human populations

**FIGURE 2:**
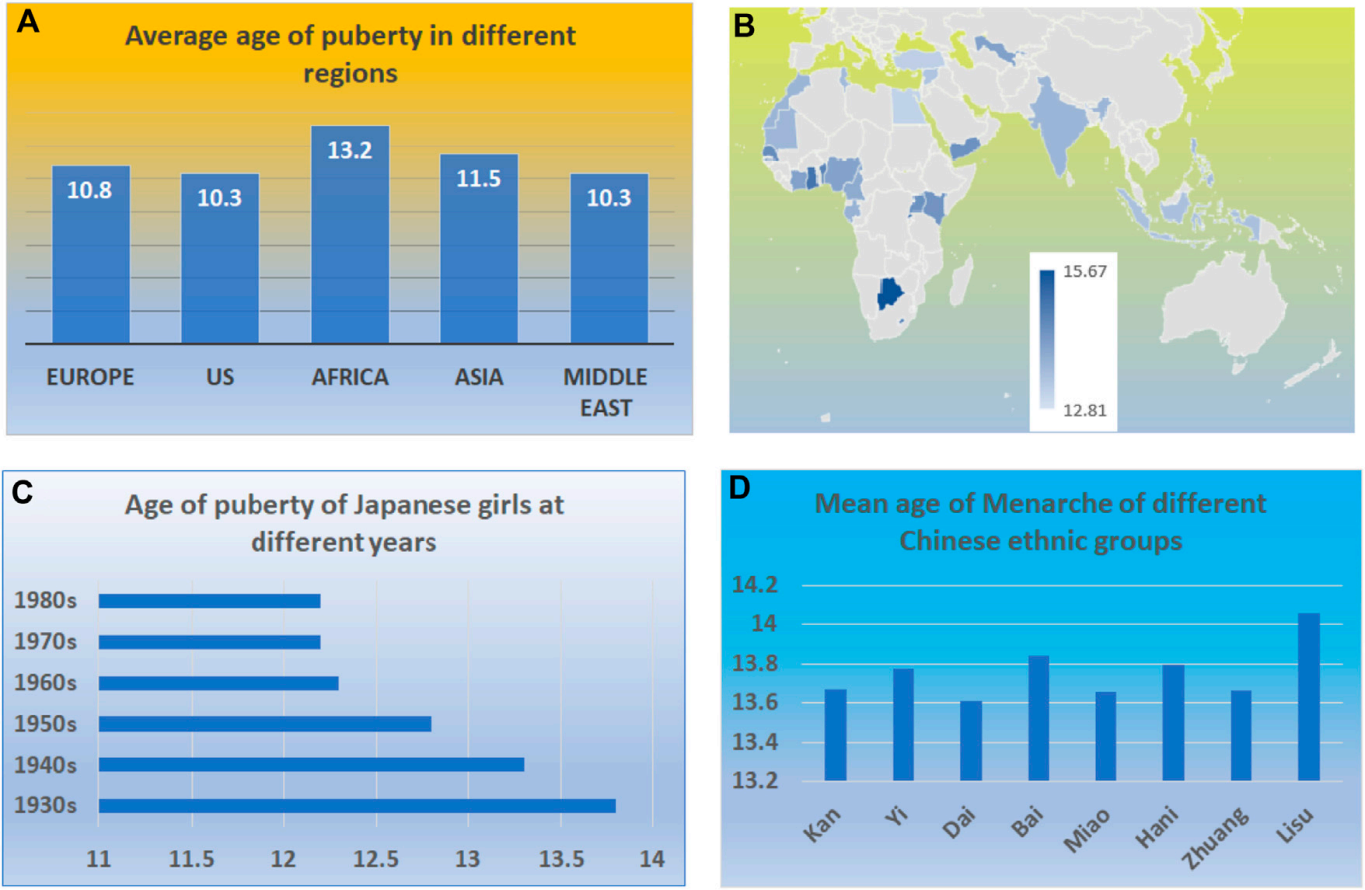
Variation of ages at puberty in the human population. **(A)** The variations in ages of puberty among different continents. **(B)** The variations in the ages of puberty among different countries among middle- and low-income countries. **(C)** The variations in the ages of puberty among Japanese girls in different years. **(D)** The variations in the ages of puberty among different Chinese ethnic groups.

It is well known that there are variations in the age of puberty among races, geographical regions, countries, and over time (Figure 2) (Thomas et al., 2001; Al-Sahab et al., 2012). However, many epidemic studies on trends and incidence of childhood diseases use the same age range in the comparison data over decades (Thomas et al., 2001; Gu, 2022b). The study on the trends in the incidence of childhood cancer statistically analyzed the incidence of cancer in Canada using samples under the age of 14 (Mitra et al., 2012). The population at the age of 14 in 1992 may represent different life stages of the 14-year-old population in 2006. (Al-Sahab et al., 2012).

### 2.8 Trends and variation of puberty among different regions

The variation of puberty can be demonstrated by the girl’s age at puberty. Figure 2 demonstrates the variation in the ages of puberty among regions, countries, and the same population (Eckert-Lind et al., 2020; Leone and Brown, 2020; Wu et al., 2022). The variation exists among not only racial groups but also the regions and countries (Figures 2A–D). One variation is the regional and racial differences (Figures 2A,D). For example, the median age for a healthy girl who attained Tanner breast stage 2 in Europe, the US, Africa, Asia, and the Middle East were from 9.8 to 10.8, 8 to 10.3, 10.1 to 13.2, 8.9 to 11.5, and 9.7–10.3 years, respectively (Eckert-Lind et al., 2020).

The other difference exists within the same region (Figure 2C). For example, within low-income and middle-income countries, there is a great variation in the age at menarche. In Colombia, the average age of menarche is 12.81, while in Botswana, the average age is 15.67 (Leone and Brown, 2020). Living conditions and environmental factors contribute to the body’s growth; in particular, food supplies and nutrients may have led to such a variation. It has been reported that both fetal and childhood famine exposure, especially in late childhood, were positively associated with increased age at menarche (Wu et al., 2022).

The other variation is the longitudinal variation. There is a trend that the age of puberty decreases worldwide (Pathak et al., 2014; Bräuner et al., 2020). For example, a recent study reports that Denmark’s annual incidence of central precocious puberty (CPP) and normal variant puberty (PT) has substantially increased in the last 20 years. The incidence rate for CPP per 10,000 girls 20 years ago and late was 13.7 (95% CI, 9.3–18.2) and recently was 14.2 (95% CI, 4.6–23.9). Similarly, the mean age at menarche among Indian women in 2005 declined by 3 months from women born prior to 1955–1964 (Pathak et al., 2014).

Most importantly, there is significant variation in the age of puberty among the same population making it more difficult to generalize based on age (Ohlsson et al., 2019; Aris et al., 2022) (Figure 2E). Complex genetic, environmental, and nutritional factors can influence normal puberty timing, precocious puberty, and delayed puberty (Aris et al., 2022). The standard variation of puberty age among girls in a country can be as much as 1.5 years (Hirokawa et al., 1992). Similarly, the age of puberty in boys is also decreased. Ohlsson et al. reported that age at peak height velocity in Swedish boys born from 1947 to 1996 was 1.5 months earlier for every decade increase in the birth year (Ohlsson et al., 2019). Aris et al. studied the early-life growth and age at pubertal onset among boys and girls in US children. They found that faster gains in weight, length or height, or body mass index in early life were associated with earlier pubertal onset (Aris et al., 2022), again suggesting that nutrition leads to variation in ages of puberty.

### 2.9 The trends and variation of the ages of menopause around the world

**FIGURE 3:**
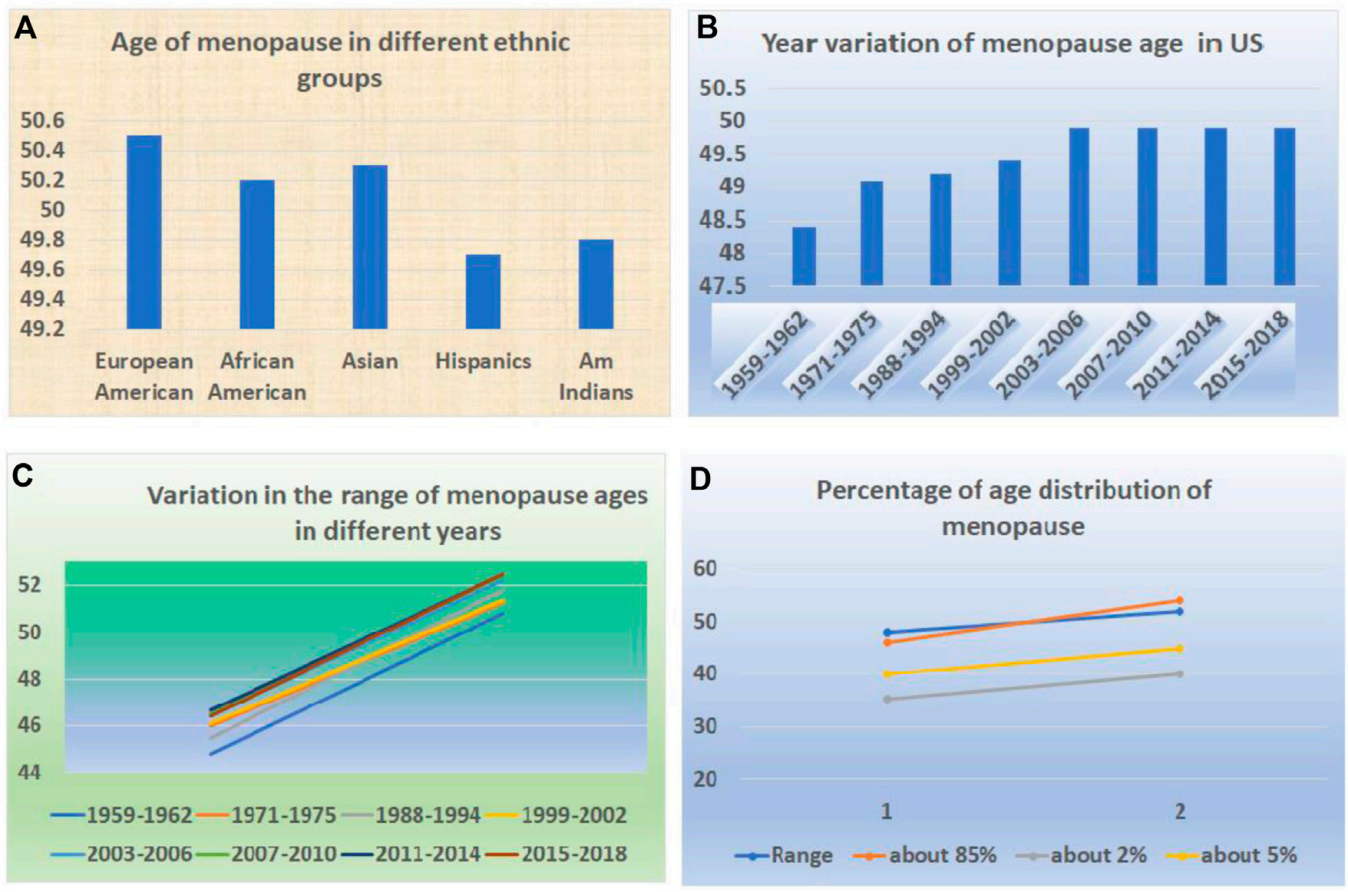
Variation of ages at menopause in the human population. **(A)** The variations in ages of menopause among different races and ethnicities. **(B)** The variations in ages of menopause in different years. **(C)** The variations in the range of ages of menopause in different years. **(D)** The different percentages of menopause ages among the same population.

Like puberty, the age of menopause in women varies greatly in similar ways (Aris et al., 2022). It varies among the geographic regions and races or ethnicities (Figure 3A). For example, a systematic review and meta-analysis on the 44 community-based studies across six continents concluded that the average ages at natural menopause among African, Latin American, Asian, Middle Eastern, European, Australian, and the United States women were 48.38, 47.24, 48.75, 47.37, 50.54, 51.25 and 49.11, respectively (Eckert-Lind et al., 2020) (Figure 3B).

Within the same region or area, there is a variation (Figure 3C). For example, within Asian countries, there is variation ranging from 46.70 to 50.10. Within European countries, the range is from 49.81 to 51.30. Within Middle Eastern countries, it ranged from 46.24 to 48.30 (Manotas et al., 2022).

The change in the trend for menopause is in the opposite direction (Figure 3D). Instead of downward, it has been in an upward direction (Schoenaker et al., 2014). For example, from 1959–1962 to 2015–2018, the mean age of natural menopause increased from 48.4 years to 49.9 years (Appiah et al., 2021). A study also indicated that age at menopause across Europe is shifting toward higher ages (Dratva et al., 2009).

In addition to the genetic variations and racial differences, there are enormous factors that influence the age at menopause (Dratva et al., 2009). These factors include the lifestyle and the living environment such as income and employment, physical and psychological health, family, work, social network, and cultural educational levels. (Zou et al., 2021). Thus, within the same country or region, variations exist in the age of menopause among women (Ringa, 2000; Carty et al., 2013).

## 3 Recommendations-the needs and benefits in the application of life stages in pharmacology

Life stages should be given consideration in all aspects of medicine. For historical reasons, life stages have been left out as an essential characteristic in medical practice. To integrate the life stage in medicine, the work should start at the very beginning, with drug testing and clinical trials, and through drug labeling and medical practice.

### 3.1 Subjects in clinical trials

Currently, only a few clinical trials on hormone therapies include puberty or menopause as selection criteria or patient characterization. The majority of clinical trials, such as the vaccine trials, use different ages but do not list life stages in the selection criteria of research subjects. For example, the trial on the COVID vaccine is divided into a range of ages, such as the age group of 5–11 years (Di Fusco et al., 2022; Kim et al., 2022). In the clinical trials of cancer patients, age groups have been widely used as the general rule to describe the response to the treatment, either for children or adults (Kang et al., 2018; Porter et al., 2022).

**FIGURE 4:**
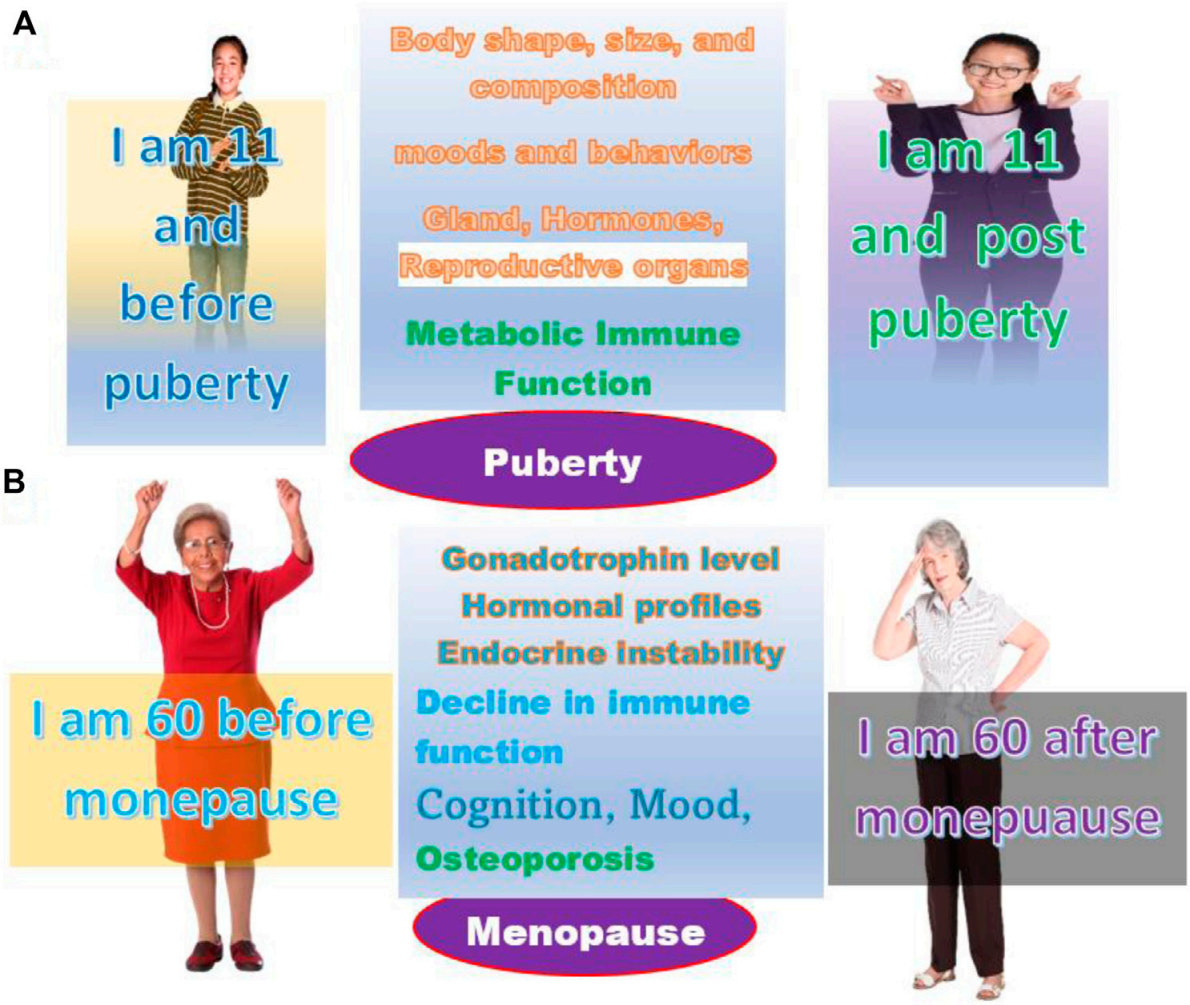
The age and life stage are not always the same. **(A)** Persons of the same age may be at the life stage of before or after puberty. **(B)** Persons of the same age may be at the life stage before or after menopause.

In reality, there are tremendous changes before and after these life stages. For a girl, before and after puberty, the differences are enormous in terms of the increase of cortisol, including energy expenditure, body growth, motor behavior, physical activity, metabolism, and most importantly, the immunological response or immune reaction. Because of this wide spectrum of changes, the response before and after puberty to most medical treatments, such as medicine or surgery, will not be the same. It is most likely that two girls at the same age, for example, at the age of 11, the one before puberty, will react to a testing drug, such as the COVID-19 vaccine, stronger and more rapidly than the one after puberty (Figure 4A).

Opposite to puberty, women after menopause will decrease their immune reaction level partially because the hormone levels will remain at a constant low level. The changes will make women vulnerable to many conditions, including osteoporosis, cardiovascular disease, and depression. These physiological, metabolic, and immunological changes lead to a different response to the majority of medical treatments than before menopause (Figure 4B).

**FIGURE 5:**
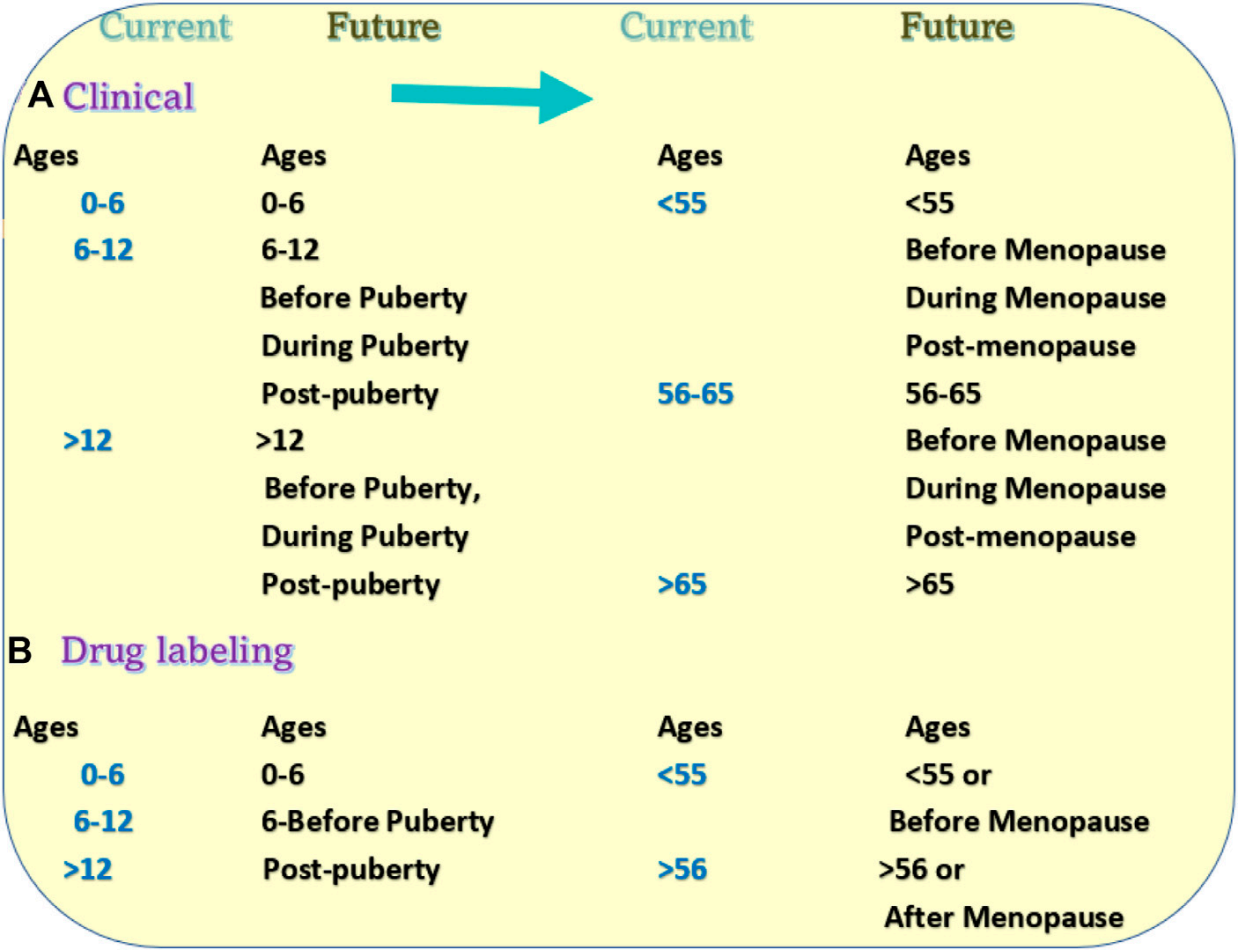
Proposed changes in clinical trials and drug labeling. **(A)** Current patient grouping and proposed grouping methods in the research subjects of clinical trials. **(B)** Current age groups in the drug direction and proposed new direction methods in the drug labeling.

Accordingly, adding life stages into patients’ information will enhance most clinical trials’ accuracy. In clinical drug trials, adding the life stages of the study subjects will enhance the accuracy of comparative data from different areas or countries because populations from different geographical areas of similar ages maybe not be in the same life stages. Within the population of the same country or regions, data from life stages will explain the efficacy of the tested medical treatment and the dosages in different life stages. (Figure 5A) (Torres-Mejía et al., 2005).

### 3.2 Drug labels

Similarly, the current labels for most drugs do not have instructions for drug usage according to the life stages. Instead, most drug labels divided usages and dosages based on age groups such as between 6 and 10, or under 11. Such a drug labeling system has its historical reason because the life stage has never been used or required as one category in drug usage and labeling.

Such a labeling system has multiple problems with drug usage. The first problem is the generalization of the age dosage. For example, the directions of one drug label divide the usage into two categories “adults and children 12 years and over” and “children under 12 years”. For category one, the direction may instruct the patient to “take 2 tablets every 4–6 h” For the second category, it may put “take 1 tablet ever 4 to 6 houses”. Such labeling can be seen in many drugs. At the age of 12, a girl may or may not be past puberty. The difference between 1 and 2 tablets may significantly affect the effectiveness of treatment (Figure 5B).

The other problem is that the dosage effect on the same age may change years later. For example, 20 years ago, a country’s average puberty age may have been 14 years old. At that time, the data on the effect of a drug from a clinical trial at the age of 13 years would have been based on girls before puberty. However, 20 years later, the average age of puberty in the same country is 12 years old. Thus, the dosage may need to be changed. Similarly, such data from one ethnic population may not be the same in another ethnic group.

Therefore, drug labels may benefit the patients, especially when the ages are labeled around puberty or menopause. Because of the differences in physiological and disease status during different life stages, it is essential to define these life stages accurately. Puberty and menopause are the visible phenotypes through these life stages; considerable future studies on the classification of different life stages are needed.

### 3.3 Drugs in clinical practice

In the medical practice, the question of puberty and menopause should not be only limited to the question asked by an endocrinologist and nurses but also by other physicians. Most importantly, while physicians and nurses talk with a girl about puberty, growth, body, and emotional changes, they should also carefully consider the dosages along with these changes for a girl receiving treatment for a disease. Similarly, this medical practice should be incorporated stages before and after the menopause stages. Guidance for physician practices in different clinical instructions should be clearly written. Unfortunately, most of these are currently lacking.

### 3.4 Male life stages

Males also have different life stages, but different criteria may be used to define these stages. Although men have the same life stages and the changes are similar to those of women in many respects, the changes in hormone levels, types, metabolic pathways, and immune reactions differ. Whether these differences affect the medical treatment will be varied based on diseases and many other conditions.

What is the same between men and women are the vast differences between different life stages. Therefore, for the clinical trials, drug labels, and medical practice, there will be the differences among life stages and sex differences. There will be tremendous work ahead to understand these differences. However, these differences are essential for personalized medicine, enhancement of human health, and the necessary improvement of disease treatment.

## 4 Conclusion: The life stage is an essential parameter in pharmacology and medicine

As discussed above, the reasons for the changes in dosages at puberty are 1). The majority of diseases occur differently before and after puberty. 2). There are dramatic changes in the immune response before and after puberty. 3). Response to drug treatment is different because of the changes in the immune system and hormone levels. 4). Currently, the majority of drugs have instructions for different dosages based on age, mostly between 12 and 14. Such a label intends to provide different doses based on the different life stages. However, the most significant change in body maturity and the immune response is between the times before and after puberty. 5). No matter influenced by which factors, life stages such as puberty and menopause are the indication of significant changes in human development. And 6). Because the ages of puberty among individuals or ethnic groups are different, instruction of doses is better based on puberty rather than ages.

For menopause, drug dose changes based on menopause are better than based on age. It is well known that after menopause, the hormone levels and immune responses are significantly decreased. Therefore, drug dosage after menopause should be different from that before menopause. A good example is the different dosages used in the vaccination among the aged population and others. The response to vaccination in the aged population is much weak than in the other populations. Similar to early puberty, a prolongation of menopause age will affect the drug’s effectiveness. In general medical practice, drug dosages are higher in the aged population than in the young population, based on the fact that immune response in the aged population are weak. However, significant changes in the immune response occur during menopause. After menopause, the immune response reduces dramatically. When a premenopausal person received the dosage for an aged person, it may bring a much higher degree of responses by this person or the complications of overdosage.

Evidently, the life stage is an essential feature of the life cycle of humankind. Different life stages have entirely different physiological and pathological characterizations, and the time and period of these life stages vary from individual to individual. In addition to other characterizations of a person such as race, gender, and body weight, life stage is an important feature that should be included in clinical trials, drug usage, and clinical practice as a key part of personalized medicine. Because of the existing variation of the life stage, age dosages of drugs and practice guidelines do not reflect the life stage of patients. We issue this call to incorporate the life stages into all aspects of medicine.

Correctly applying life stages in medicine faces many challenges. There are differences between inner physiological changes and phenotypic signs of body changes. Considerable research must be conducted before clearly defining the turning point of puberty and menopause. The application of life stages into clinical trials, drug labeling, and clinical practice involves the medical field but also relates to social and economic issues, including health regulation, insurance policy, and law. Such a change needs to be initiated with the effort of a whole society.

To initiate such a change, this paper will serve as a call for awareness of the importance and the urgency for tcorrecting such a problem in our clinical practice and drug usage by the biomedical community and clinicians. We need consensus from clinicians, basic researchers, and community leaders, perhaps with more evidence and discussion. When such a consensus is reached, guidelines for such a change from relevant associations such as endocrinology can be written. Rules and regulations by policymakers from relevant organizations and governments may follow. WE sincerely hope that this day will come soon.

## References

[B1] Al-Sahab B. Ardern C. I. Hamadeh M. J. Tamim H. (2012). Age at menarche and current substance use among Canadian adolescent girls: Results of a cross-sectional study. BMC Public Health 12, 195. 10.1186/1471-2458-12-195 22424106 PMC3323411

[B2] Appiah D. Nwabuo C. C. Ebong I. A. Wellons M. F. Winters S. J. (2021). Trends in age at natural menopause and reproductive life span among US women, 1959-2018. JAMA 325 (13), 1328–1330. 10.1001/jama.2021.0278 33821908 PMC8025101

[B3] Aris I. M. Perng W. Dabelea D. Ganiban J. M. Liu C. Marceau K. (2022). Analysis of early-life growth and age at pubertal onset in US children. JAMA Netw. Open 5 (2), e2146873. 10.1001/jamanetworkopen.2021.46873 35119461 PMC8817204

[B4] Bräuner E. V. Busch A. S. Eckert-Lind C. Koch T. Hickey M. Juul A. (2020). Trends in the incidence of central precocious puberty and normal variant puberty among children in Denmark, 1998 to 2017. JAMA Netw. Open 3 (10), e2015665. 10.1001/jamanetworkopen.2020.15665 33044548 PMC7550972

[B5] Carty C. L. Spencer K. L. Setiawan V. W. Fernandez-Rhodes L. Malinowski J. Buyske S. (2013). Replication of genetic loci for ages at menarche and menopause in the multi-ethnic Population Architecture using Genomics and Epidemiology (PAGE) study. Hum. Reprod. 28 (6), 1695–1706. 10.1093/humrep/det071 23508249 PMC3657124

[B6] Chakraborty D. Rangamani S. Kulothungan V. Chaturvedi M. Stephen S. Das P. (2018). Trends in incidence of ewing sarcoma of bone in India - evidence from the national cancer registry programme (1982-2011). J. Bone Oncol. 12, 49–53. 10.1016/j.jbo.2018.04.002 30237969 PMC6142187

[B7] Collaborative Group on Hormonal Factors in Breast Cancer (2012). Menarche, menopause, and breast cancer risk: Individual participant meta-analysis, including 118 964 women with breast cancer from 117 epidemiological studies. Lancet. Oncol. 13 (11), 1141–1151. 10.1016/S1470-2045(12)70425-4 23084519 PMC3488186

[B8] Davis S. R. Lambrinoudaki I. Lumsden M. Mishra G. D. Pal L. Rees M. (2015). Nat. Rev. Dis. Prim. 1, 15004. 10.1038/nrdp.2015.4 27188659

[B9] Day F. R. Thompson D. J. Helgason H. Chasman D. I. Finucane H. Sulem P. (2017). Genomic analyses identify hundreds of variants associated with age at menarche and support a role for puberty timing in cancer risk. Nat. Genet. 49 (6), 834–841. 10.1038/ng.3841 28436984 PMC5841952

[B10] Di Fusco M. Vaghela S. Moran M. M. Lin J. Atwell J. E. Malhotra D. (2022). COVID-19-associated hospitalizations among children less than 12 years of age in the United States. J. Med. Econ. 25 (1), 334–346. 10.1080/13696998.2022.2046401 35293285

[B11] Dratva Julia Gómez Real Francisco Schindler Christian Ackermann-Liebrich Ursula Gerbase Margaret W. Probst-Hensch Nicole M. (2009). Is age at menopause increasing across Europe? Results on age at menopause and determinants from two population-based studies. Menopause 16 (2), 385–394. 10.1097/gme.0b013e31818aefef 19034049

[B12] Eckert-Lind C. Busch A. S. Petersen J. H. Biro F. M. Butler G. Brauner E. V. (2020). Worldwide secular trends in age at pubertal onset assessed by breast development among girls: A systematic review and meta-analysis. JAMA Pediatr. 10, e195881. 10.1001/jamapediatrics.2019.5881 PMC7042934 32040143

[B13] Filatova S. Upadhyaya S. Kronström K. Suominen A. Chudal R. Luntamo T. (2019). Time trends in the incidence of diagnosed depression among people aged 5-25 years living in Finland 1995-2012. Nord. J. Psychiatry 73 (8), 475–481. 10.1080/08039488.2019.1652342 31443615

[B14] Gameiro C. M. Romão F. Castelo-Branco C. (2010). Menopause and aging: Changes in the immune system--a review. Maturitas 67 (4), 316–320. 10.1016/j.maturitas.2010.08.003 20813470

[B15] Gu W. (2022). Healthy long-lived human beings-working on life stages to break the limitation of human lifespans. Biol. (Basel) 11 (5), 656. 10.3390/biology11050656 PMC9137948 35625384

[B16] Gu W. (2022). It is time to work on the extension of body growth and reproductive stages. Rejuvenation Res. 25 (2), 110–115. 10.1089/rej.2022.0017 35293249

[B17] Hirokawa K. Utsuyama M. Kasai M. Kurashima C. (1992). Aging and immunity. Acta Pathol. Jpn. 42 (8), 537–548. 10.1111/j.1440-1827.1992.tb03103.x 1449050

[B18] Kang H. J. Loftus S. DiCristina C. Green S. Pong A. Zwaan C. M. (2018). Aprepitant for the prevention of chemotherapy-induced nausea and vomiting in paediatric subjects: An analysis by age group. Pediatr. Blood Cancer 65 (10), e27273. 10.1002/pbc.27273 29893452

[B19] Kim C. Yee R. Bhatkoti R. Carranza D. Henderson D. Kuwabara S. A. (2022). COVID-19 vaccine provider access and vaccination coverage among children aged 5-11 Years - United States, november 2021-january 2022. MMWR. Morb. Mortal. Wkly. Rep. 71 (10), 378–383. 10.15585/mmwr.mm7110a4 35271559 PMC8911999

[B20] Leone T. Brown L. J. (2020). Timing and determinants of age at menarche in low-income and middle-income countries. BMJ Glob. Health 5 (12), e003689. 10.1136/bmjgh-2020-003689 PMC7733094 33298469

[B21] Liu Z. Mao J. Wu X. Xu H. Wang X. Huang B. (2016). Efficacy and outcome predictors of gonadotropin treatment for male congenital hypogonadotropic hypogonadism: A retrospective study of 223 patients. Med. Baltim. 95 (9), e2867. 10.1097/MD.0000000000002867 PMC4782854 26945370

[B22] Lobo R. A. (2017). Hormone-replacement therapy: Current thinking. Nat. Rev. Endocrinol. 13 (4), 220–231. 10.1038/nrendo.2016.164 27716751

[B23] Lucas-Herald A. Bertelloni S. Juul A. Bryce J. Jiang J. Rodie M. (2016). The long-term outcome of boys with partial androgen insensitivity syndrome and a mutation in the androgen receptor gene. J. Clin. Endocrinol. Metab. 101 (11), 3959–3967. Epub 2016 Jul 12. 10.1210/jc.2016-1372 27403927 PMC5095251

[B24] Manotas M. C. González D. M. Céspedes C. Forero C. Rojas Moreno A. P. (2022). Genetic and epigenetic control of puberty. Sex. Dev. 16 (1), 1–10. 10.1159/000519039 PMC8820423 34649256

[B25] Mitra D. Shaw A. K. Hutchings K. (2012). Trends in incidence of childhood cancer in Canada, 1992-2006. Chronic Dis. Inj. Can. 32 (3), 131–139. 10.24095/hpcdp.32.3.03 22762899

[B26] Muka T. Oliver-Williams C. Kunutsor S. Laven J. S. Fauser B. C. Chowdhury R. (2016). Association of age at onset of menopause and time since onset of menopause with cardiovascular Outcomes, intermediate vascular traits, and all-cause mortality: A systematic review and meta-analysis. JAMA Cardiol. 1 (7), 767–776. 10.1001/jamacardio.2016.2415 27627190

[B27] Ohlsson C. Bygdell M. Celind J. Sondén A. Tidblad A. Sävendahl L. (2019). Secular trends in pubertal growth acceleration in Swedish boys born from 1947 to 1996. JAMA Pediatr. 173 (9), 860–865. 10.1001/jamapediatrics.2019.2315 31329245 PMC6647355

[B28] Ohlsson C. Bygdell M. Nethander M. Kindblom J. M. (2020). Early puberty and risk for type 2 diabetes in men. Diabetologia 63 (6), 1141–1150. 10.1007/s00125-020-05121-8 32201902 PMC7228987

[B29] Pathak P. K. Tripathi N. Subramanian S. V. (2014). Secular trends in menarcheal age in India-evidence from the Indian human development survey. PLoS One 9 (11), e111027. 10.1371/journal.pone.0111027 25369507 PMC4219698

[B30] Porter A. B. Liu H. Kohli S. Cerhan J. L. Sloan J. McMurray R. P. (2022). Efficacy of treatment with armodafinil for cancer-related fatigue in patients with high-grade glioma: A phase 3 randomized clinical trial. JAMA Oncol. 8 (2), 259–267. 10.1001/jamaoncol.2021.5948 34882169 PMC8662535

[B31] Qiu C. Chen H. Wen J. Zhu P. Lin F. Huang B. (2013). Associations between age at menarche and menopause with cardiovascular disease, diabetes, and osteoporosis in Chinese women. J. Clin. Endocrinol. Metab. 98 (4), 1612–1621. 10.1210/jc.2012-2919 23471979

[B32] Ringa V. (2000). Menopause and treatments. Qual. Life Res. 9 (6), 695–707. 10.1023/a:1008913605129

[B33] Schoenaker D. A. Jackson C. A. Rowlands J. V. Mishra G. D. (2014). Socioeconomic position, lifestyle factors and age at natural menopause: A systematic review and meta-analyses of studies across six continents. Int. J. Epidemiol. 43 (5), 1542–1562. 10.1093/ije/dyu094 24771324 PMC4190515

[B34] Thomas F. Renaud F. Benefice E. de Meeus T. Guegan J. F. (2001). International variability of ages at menarche and menopause: Patterns and main determinants. Hum. Biol. 73, 271–290. 10.1353/hub.2001.0029 11446429

[B35] Tondo L. Pinna M. Serra G. De Chiara L. Baldessarini R. J. (2017). Age at menarche predicts age at onset of major affective and anxiety disorders. Eur. Psychiatry 39, 80–85. Epub 2016 Dec 16. 10.1016/j.eurpsy.2016.08.001 27992810

[B36] Torres-Mejía G. Cupul-Uicab L. A. Allen B. Galal O. Salazar-Martínez E. Lazcano-Ponce E. C. (2005). Comparative study of correlates of early age at menarche among Mexican and Egyptian adolescents. Am. J. Hum. Biol. 17 (5), 654–658. 10.1002/ajhb.20420 16136537

[B37] Wang L. Cao Y. Ren M. Chen A. Cui J. Sun D. (2017). Sex differences in hazard ratio during drug treatment of non-small-cell lung cancer in major clinical trials: A focused data review and meta-analysis. Clin. Ther. 39 (1), 34–54. 10.1016/j.clinthera.2016.12.008 28069259

[B38] Wang L. Liu F. Li J. Ma L. Feng H. Liu Q. (2021). From anti-PD-1/PD-L1 to CTLA-4 and to MUC1-is the better response to treatment in smokers of cancer patients drug specific? J. Pers. Med. 11 (9), 914. 10.3390/jpm11090914 34575691 PMC8471889

[B39] Watson S. Caster O. Rochon P. A. den Ruijter H. (2019). Reported adverse drug reactions in women and men: Aggregated evidence from globally collected individual case reports during half a century. EClinicalMedicine 17, 100188. 10.1016/j.eclinm.2019.10.001 31891132 PMC6933269

[B40] Wu X. Bao L. Du Z. Liu X. Liao W. Kang N. (2022). Secular trends of age at menarche and the effect of famine exposure on age at menarche in rural Chinese women. Ann. Hum. Biol. 49 (1), 35–40. Epub 2022 Mar 11. 10.1080/03014460.2022.2041092 35139699

[B41] Ysrraelit M. C. Correale J. (2019). Impact of sex hormones on immune function and multiple sclerosis development. Immunology 156 (1), 9–22. 10.1111/imm.13004 30222193 PMC6283654

[B42] Zou P. Waliwitiya T. Luo Y. Sun W. Shao J. Zhang H. (2021). Factors influencing healthy menopause among immigrant women: A scoping review. BMC Womens Health 21 (1), 189. 10.1186/s12905-021-01327-z 33957910 PMC8101137

